# PFOS Induces Behavioral Alterations, Including Spontaneous Hyperactivity That Is Corrected by Dexamfetamine in Zebrafish Larvae

**DOI:** 10.1371/journal.pone.0094227

**Published:** 2014-04-16

**Authors:** Stefan Spulber, Pascal Kilian, Wan Norhamidah Wan Ibrahim, Natalia Onishchenko, Mazhar Ulhaq, Leif Norrgren, Sara Negri, Marcello Di Tuccio, Sandra Ceccatelli

**Affiliations:** 1 Dept of Neuroscience, Karolinska Institutet, Stockholm, Sweden; 2 Department of Biology, Faculty of Science, Universiti Putra Malaysia, Serdang, Selangor, Malaysia; 3 Department of Biomedicine and Veterinary Public Health, Swedish University of Agricultural Sciences, Uppsala, Sweden; 4 Environmental Research Center, Salvatore Maugeri Foundation – IRCCS, Pavia, Italy; Institut Curie, France

## Abstract

Perfluorooctane sulfonate (PFOS) is a widely spread environmental contaminant. It accumulates in the brain and has potential neurotoxic effects. The exposure to PFOS has been associated with higher impulsivity and increased ADHD prevalence. We investigated the effects of developmental exposure to PFOS in zebrafish larvae, focusing on the modulation of activity by the dopaminergic system. We exposed zebrafish embryos to 0.1 or 1 mg/L PFOS (0.186 or 1.858 µM, respectively) and assessed swimming activity at 6 dpf. We analyzed the structure of spontaneous activity, the hyperactivity and the habituation during a brief dark period (visual motor response), and the vibrational startle response. The findings in zebrafish larvae were compared with historical data from 3 months old male mice exposed to 0.3 or 3 mg/kg/day PFOS throughout gestation. Finally, we investigated the effects of dexamfetamine on the alterations in spontaneous activity and startle response in zebrafish larvae. We found that zebrafish larvae exposed to 0.1 mg/L PFOS habituate faster than controls during a dark pulse, while the larvae exposed to 1 mg/L PFOS display a disorganized pattern of spontaneous activity and persistent hyperactivity. Similarly, mice exposed to 0.3 mg/kg/day PFOS habituated faster than controls to a new environment, while mice exposed to 3 mg/kg/day PFOS displayed more intense and disorganized spontaneous activity. Dexamfetamine partly corrected the hyperactive phenotype in zebrafish larvae. In conclusion, developmental exposure to PFOS in zebrafish induces spontaneous hyperactivity mediated by a dopaminergic deficit, which can be partially reversed by dexamfetamine in zebrafish larvae.

## Introduction

Perfluorinated compounds (PFCs) are a family of chemical compounds that are very stable in the environment due to the carbon-fluorine bonds (reviewed in [1]). Perfluorooctane sulfonate (PFOS) is a member of the PFCs family that has been used in a wide range of industrial and commercial applications for the past 50 years and is the most commonly found in a large number of biomonitoring samples from the human general population and wildlife [2]–[4]. In the human population, the main route of exposure to PFOS is believed to be through consumption of contaminated food, such as fish, aquatic invertebrates, marine mammals, and other composite food, as well as contaminated drinking water [5]–[7]. The elimination half-life of PFOS (5.4 years in humans [8]) and its persistence in the environment raise concerns about the potential adverse impact on human health, particularly when the exposure occurs during early developmental stages (see [9]). PFOS has been detected in the umbilical cord and in maternal milk, and there is epidemiological evidence of dose-dependent increase in the risk of adverse birth outcomes [10]–[12]. Prenatal exposure to PFOS has not been found to be associated to neurodevelopmental deficits in children up to the age of 7 [8], [13], [14]. However, two cross-sectional studies report an association between blood levels of PFOS and impulsivity [15] and the prevalence of attention deficit hyperactive disorder (ADHD) [16] in children aged 9–11 and 12–15, respectively.

In rodents, developmental toxicity studies on the effects of PFOS have revealed reduction of fetal weight, reduced neonatal survival, defects in the peripheral nervous system, and behavioral alterations [17]–[20]. In addition, after maternal exposure, PFOS accumulates in the developing brain before the formation of the blood brain barrier (BBB) [21], and can also cross the BBB to a certain extent [22]–[24]. *In vitro* studies have shown that PFOS enhances the differentiation of cortical neural stem cells towards neuronal and oligodendroglial phenotypes [25], and that the neuronal differentiation induced in PC12 cells is shifted towards a cholinergic, rather than a dopaminergic phenotype [26].

Most developmental neurotoxicity data available on PFCs were generated using rodents as animal models. Zebrafish (*Danio rerio*) has emerged as an alternative animal model for pharmacological and toxicological studies due to the conserved neurotransmitter systems and molecular signaling pathways associated to behavioral responses (reviewed in [27], [28]). The aim of this study was to investigate the effects of developmental exposure to PFOS on the activity pattern in zebrafish larvae, with special focus on the modulation of swimming activity by the dopaminergic system. To this end we exposed zebrafish embryos to PFOS at concentrations that do not increase the mortality or the incidence of embryonic malformations [29], [30]. We analyzed the behavior at larval stage, with special focus on the pattern of spontaneous activity, as well as on the transient hyperactivity induced by a dark pulse (visual motor response, VMR) or by a vibrational stimulus (startle response). In light of the findings in zebrafish larvae, we further analyzed historical behavioral data from rodents [19] and found similar phenotypes in the spontaneous locomotion in mice exposed to PFOS during early developmental stages. Altogether, our data indicate that developmental exposure to PFOS results in behavioral alterations and a central dopamine signaling deficit which provide experimental evidence supporting the reported epidemiological association between exposure to PFOS and impulsivity [15], [16].

## Materials and Methods

### Chemicals

PFOS (heptadecafluorooctanesulfonic acid potassium salt, CAS 2795-39-3, purity >98%) was purchased from Sigma-Aldrich, dissolved in DMSO (1 mg/mL), and stored at 4°C as stock solution for exposure in zebrafish embryos. For exposing pregnant mouse females, PFOS was dissolved in pure ethanol immediately before administration on palatable pieces of food (cookie bits that were readily consumed by the animals).

### Animals: exposure and behavioural tests

All experimental procedures were performed in agreement with the Swedish animal protection legislation and European regulations, and were approved by the local Animal Ethics Committee (Stockholms Norra djurförsöksetiska nämnd).

#### Zebrafish

Wildtype AB zebrafish embryos were obtained from the zebrafish core facility at Karolinska Institutet. Breeding groups of adult fish (3 males and 2 females) were housed together overnight in 10 L spawning tanks containing environmental enrichment (commercially available aquaria decoration made of non-toxic plastic). Thirty minutes after turning the light on (9:00 a.m.), the fertilized eggs were collected and stored at 28.5°C until further processing. The eggs were washed twice with fresh E3 water (5 mM NaCl, 0.17 mM KCl, 0.33 mM CaCl_2_, 0.33 mM MgSO_4_; 0.05% methylene blue; pH 7.4) in order to remove all debris. This procedure was performed on a clean open lab bench, at room temperature, under constant illumination (approximately 500 lx). Stock PFOS was further diluted in DMSO to yield a final concentration of 0.1% DMSO in the rearing water. The zebrafish embryos were exposed to PFOS concentrations of either 0.1, or 1 mg/L (0.186 or 1.858 µM, respectively). The doses were selected based on our experience and earlier reports [30]–[32] to avoid a significant increase in the rate of embryonal malformations and lethality. Control embryos were exposed to 0.1% DMSO in E3 water. The exposure to PFOS was initiated about 2 hours post fertilization (hpf). The zebrafish larvae were then plated and maintained individually in 48-well plates (cylindrical wells, 10 mm inner diameter; VWR, Leuven, Belgium) in 750 µl E3 water at 28°C in a 10:14 h dark-light cycle (light on at 9 a.m.; 300 lx intensity, daylight-matching spectrum white light) until behavioral testing at 6 days post fertilization (dpf). The exposure followed a static, non-replacement regime (*i.e.* the rearing water was not refreshed or replaced throughout the experiment, according to OECD guidelines [33], [34] and earlier reports [30], [32], [35]). All treatments were present in each plate, and the larvae were distributed such that an equal proportion from each group would be placed in wells at the periphery of the plate. The mortality, successful hatching, and the occurrence of embryonal malformations as assessed at the 24 (developmental failure/coagulation, light-induced coiling movements), 48 (developmental failure/coagulation, blood circulation, pericardial oedema, pigmentation), 72, and 120 hpf (hatching, eye development, swimming bladder inflation, morphological abnormalities such as scoliosis or bent spine), as well as after completing the behavioural experiments (6 dpf) using an inverted microscope (Nikon Ti-S) in brightfield configuration with a 4× objective. The larvae displaying morphological abnormalities at 6 dpf were excluded from analyses.

*Determination of PFOS in zebrafish larvae by UPLC-MS/MS.* The zebrafish larvae were killed by rapid cooling in ice-cold water immediately after the behavioural recording at 6 dpf. The larvae were then collected and stored in Eppendorf tubes with a minimal amount of rearing water to preserve the integrity of the organism until further processing. All larvae from an experiment were pooled per treatment to yield a sufficient amount of tissue (22±14 mg per treatment group from 6 independent experiments). The internal dosimetry was performed by liquid chromatography coupled with mass spectrometry using an Acquity UPLC system (Waters, Milford, MA, USA) coupled with a triple quadrupole Waters TQD mass spectrometer. The samples were homogenized with acetonitrile (100 µL x 10 mg) using an ultrasonic homogenizer (Omni-Ruptor 250, Omni International). An internal standard (Perfluorononanoic acid, PFNA) was added to 100 µL of supernatant and then the samples (2 µL) were diluted 1:40 with 2 mM ammonium acetate - methanol solution (50∶50, v/v) before injection in UPLC. A calibration curve was prepared by spiking PFOS and PFNA standard to a pool of homogenized control larvae in the range from 100 to 1600 absolute ng of PFOS. Chromatographic separation was performed on an Acquity UPLC BEH C18 column (2.1×100 mm, 1.7 µm) maintained at 30°C, by gradient elution with a mixture containing variable proportions of 2 mM ammonium acetate buffer and methanol delivered at a flow rate of 0.5 mL/min. Under these conditions isomers of PFOS eluted at 1.6, 1.9, 2.1 and 2.5 minutes whereas PFNA eluted at 2.4 minutes. The total run time was 9 min. For the MS/MS detection, quantitative analysis was obtained in multiple reaction monitoring (MRM) in negative ion mode (ESI-). In particular for all the isomers of PFOS 499.0→79.8 m/z was used for quantification and 499.0 → 98.8 for confirmation; for PFNA 463.0→418.9 was selected. The limit of detection for PFOS (at a signal-to-noise ratio of 3) was 0.2 ng.

**Figure 1:**
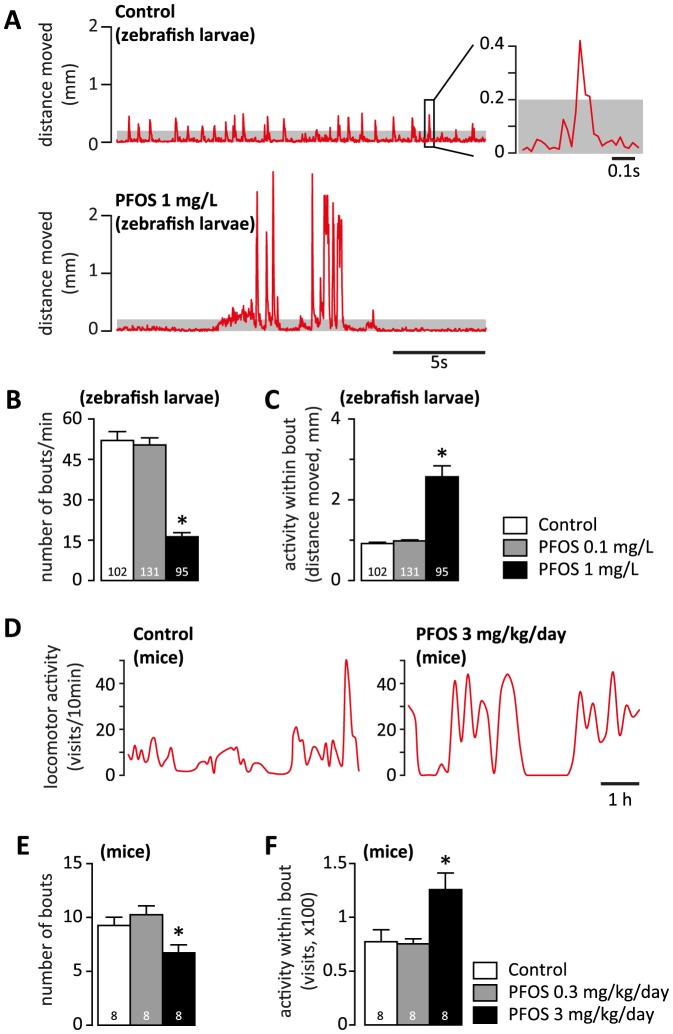
The structure of spontaneous locomotion in zebrafish larvae and mice during the active phase of the circadian cycle (light for zebrafish larvae, dark for mice). (A) Representative sequence of spontaneous activity (20 s) recorded at 6 dpf in one control and one larva exposed to 1 mg/L PFOS. In the control larva, the spontaneous activity consists of short, evenly spread bouts of swimming. In the larva exposed to 1 mg/L PFOS, the spontaneous activity consists of clusters of intense activity separated by extended periods of inactivity. Background noise (displacement below 0.2 mm/frame) shaded in gray; inset in top panel – magnification of a representative bout of spontaneous activity. (B) Quantification of frequency of spontaneous bouts of activity. Note the dramatic decrease in bout frequency induced by exposure to 1 mg/L PFOS. (C) Average distance moved during one spontaneous bout of activity. Zebrafish larvae exposed to 1 mg/L PFOS have a hyperactive phenotype characterized by a 2.5 fold higher distance swum during spontaneous bouts of activity. (D) Illustrative sequence of spontaneous locomotion during the active phase of the circadian cycle in one control and one mouse exposed to 3 mg/kg/dy PFOS during gestation. The spontaneous locomotor activity (visits) in the homecage is integrated over consecutive, non-overlapping 10 min bins and spline-interpolated for clarity. (E, F) Similar to the pattern found in zebrafish larvae, the mice exposed to 3 mg/kg PFOS display less frequent (E), but more intense (F) bouts of activity. B, C, E, F – one-way ANOVA followed by Dunnett's post-hoc test; * p<0.05 PFOS exposed vs. control. The number of independent observations is indicated at the bottom of each column in B, C, E and F.

*Behavioral tests and analyses in zebrafish larvae.* We recorded the locomotor activity of 6 dpf zebrafish larvae at 50 frames per second (fps) under constant infrared illumination using integrated high throughput systems for videotracking and environmental control (ZebraBox™, ViewPoint, Lyon, France, and DanioVision, Noldus, Wageningen, The Netherlands). The larvae were detected by automated online image analysis using a dynamic subtraction algorithm with individual frame weight of 4%. All videotracking data was visually inspected for detection accuracy before inclusion in further analyses. The raw tracking data were exported and analyzed using routines custom developed in Matlab™ R2012b environment (MathWorks Inc., Natick, MA, USA). To minimize the effect of missing samples (*e.g.* when the fish moved too fast over few frames, or too little to meet the automated detection criteria), the position of center of gravity was approximated using linear interpolation of *x* and *y* coordinates between the samples at the beginning and at the end of the missing samples sequence. For the analysis of bouts, we used a threshold of 0.2 mm/frame to filter out detection noise. A bout of activity was defined as the activity detected in a continuous sequence of frames during which the measured displacement of the center of gravity is above the threshold (see insert in Fig 1 A). Bouts separated by less than 40 ms (2 frames) were considered as a single bout. The following parameters were calculated for measuring the spontaneous activity bouts: frequency of occurrence, expressed in number of bouts per time unit (bouts/min); and average distance moved within the bout (mm).

*Spontaneous activity.* Before initiating the behavioural experiments, the temperature control system of the recording chamber (DanioVision™, Noldus) was allowed to run continuously until equilibrium was reached, and the temperature did not vary by more than 0.2°C over 5 min. The experimental workflow ensured that (1) the handling of the multi-well plate was limited to less than 30 s in total; (2) utmost care was taken to limit the vibrations transferred to the plate during handling; (3) the larvae did not experience major fluctuations in light intensity, particularly not towards sudden decrease (the light sources inside the recording chamber were on when the multi-well plate was placed inside, and the intensity of the lighting did not change except when testing the VMR). The larvae were allowed to acclimatize allowed to acclimatize for at least 15 min after transfer to the recording chamber. This procedure was found sufficient (pilot tests; not shown) for the spontaneous activity to be reliably recorded and analysed over reasonably long periods free from trends or trailing effects of changes in the environment. We estimated the average frequency of occurrence, and distance moved within spontaneous bouts over at least 3 min after the acclimatization period and prior to any further experimental challenge.

*The visual motor response (VMR)* is one of the earliest behavioral responses that can be recorded in freely moving zebrafish larvae (already at 4 dpf), and consists of a robust increase in swimming activity in response to a sudden decrease in the environmental light intensity [36]–[38]. Yet, at 4 dpf, the energy required for growth and swimming is provided by the protein-rich yolk, and although the larvae display a robust locomotor response to changes in light intensity, the spontaneous activity varies widely between individuals and does not support reliable estimations of baseline activity [39]. Therefore, the analysis of activity induced by dark pulses was performed at 6 dpf as follows: the larvae were placed in the DanioVision™ chamber and allowed to acclimatize for at least 15 min before starting the experiment (environmental temperature maintained at 28°C by means of a custom-built system for circulating temperature-controlled water; light intensity 3200–3500 lx). The baseline activity under constant illumination was recorded during the last 10 min of the acclimatization period. The larvae were then exposed to a dark pulse (10 min; light not detectable in the visible spectrum). The transition between light and dark was virtually instantaneous.

To characterize the hyperactivity induced by the dark pulse, we used two orthogonal parameters derived from the cumulative activity curve: the total locomotor activity and the index of curvature (IOC). IOC was calculated according to the formula below, as described by Fry et al. [40]: 
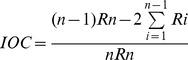
, where *R_i_* denotes the value of the *i*-th bin in the cumulative histogram. The IOC can assume values between *-(n-1)/n* and *(n-1)/n* (approaching -1 and 1, respectively, for very large *n*), where *n* is the number of observation bins (time intervals). If the activity is recorded predominantly in the beginning of the observation interval (*e.g*. if the animals habituate to the environment), IOC is negative, and the absolute value increases with the bias of distribution in activity measures. In contrast, an IOC value close to 0 indicates a uniform distribution of activity within the observation period. The rationale for calculating a parameter to describe the shape of the curve is that (1) the novelty-induced hyperactivity is a consistent finding in all animal models; (2) the locomotor activity necessarily decays with time as the animals habituate to the environment [41], [42]; and (3) the shape of the habituation curve conveys ethologically relevant information, such as the rate of habituation or acquisition of reinforcement [40]. As compared to the measures of habituation described earlier [42], [43], the IOC is a longitudinal measure of the activity and has the advantage of orthogonality in relation to global measures of activity.

*The startle response* consists of a brief increase in swimming activity (typically a single bout that is more intense than spontaneous bouts) shortly after the application of a stimulus (vibrational, acoustic, electric, or tactile), followed by a period of inactivity before resuming spontaneous activity [44] (Movie S1). The acoustic/vibrational startle response in particular develops in parallel with the auditory system and consistent behavioral responses can be recorded no sooner than 5 dpf in normal zebrafish larvae [45], [46]. We have used a custom-built system consisting of a solenoid hitting the chassis of the recording chamber. The stimulus was triggered from the videotracking software (EthoVision XT 9, Noldus, Wageningen, The Netherlands) and was synchronized with the behavioral observation. The fish were allowed to habituate to the experimental environment for 4 minutes before delivering the first stimulus. The baseline activity was recorded during the last minute of the habituation period. A series of 10 equally spaced taps (1 min intervals) were delivered during each session. A startle response was validated only if we recorded a bout of activity within 0.5 s (25 frames) after triggering the stimulus. After validation, we analyzed the following parameters: average distance moved per time unit (1 s); latency to startle (measured as the delay between the application of the stimulus and the beginning of the first subsequent bout); distance moved within the bout; inactive period following the startle response (measured as the delay between the end of the startle response and the beginning of the first next bout) (see also Figure S1). We found no evidence of habituation (decreasing number of responsive larvae, or decreasing intensity of the startle response) in our system (data not shown), and estimated group means after averaging the values for each parameter across the validated startle responses for each larva.

*Pharmacological challenges in zebrafish larvae.* Apomorphine (Apoteket, Sweden), dexamfetamine (S(+)-amfetamine sulfate, Cat. No. A-127; RBI, Natick, MA, USA), quinpirole (Tocris Bioscience, Bristol, UK), and SKF-81297 (Tocris Bioscience, Bristol, UK) were dissolved in 0.1% DMSO E3water immediately before adding to the rearing water. The pharmacological compounds were delivered in 20 µL E3 medium/well 30 min prior to recording the swimming activity. The final concentrations in the rearing water were as follows: dexamfetamine – 1 and 10 µM; apomorphine – 2.5 and 5 µM; quinpirole – 10 and 20 µM; SKF-81297 – 25 and 50 µM. The doses and the timing of behavioural recording relative to the administration of the selected compounds were set based on previous reports (for dexamfetamine, apomorphine, and quinpirole see [47], [48]) and dose-response curves tested in our lab (for SKF-81297). In particular the doses of dexamfetamine were selected so that the low dose would induce hyperactivity, and the high dose would suppress spontaneous activity in control larvae [48]. All plates were treated similarly in each experiment, *i.e.* all larvae in one plate received the same concentration of the compound, and the different concentrations were divided between plates originating from the same stock and spawning session. All experiments were repeated at least 3 times, and used fertilized eggs produced from different stocks. This design was carefully observed in order to minimize the bias of genetic background differences across stocks.

#### Historical behavioral data in mice

In light of the phenotypical alterations found in zebrafish, as well as to strengthen the relevance of zebrafish larvae as experimental model for developmental neurotoxicity, we further analyzed historical behavioral data from mice. The developmental exposure to PFOS in mice has been described earlier [19]. Briefly, pregnant C57Bl/6/Bkl dams (Scanbur BK, Sweden) received PFOS at a dose of 0.3 or 3 mg/kg/day throughout gestation (N = 6 per treatment). The locomotor activity was recorded in 8 male pups (5–6 weeks of age), randomly selected from all litters (maximum 2 pups from any litter were included). At weaning, the pups were implanted with sterile subcutaneous radio frequency identification (RFID) tags (ID-100A, Trovan Ltd., UK) under 4% isoflurane anesthesia. The transponders allowed unambiguous identification of animals throughout the experiment.

*Monitoring and analysis of spontaneous locomotion in freely moving mice.* The locomotor activity of freely moving mice was monitored using the TraffiCage™ system (NewBehavior, Zürich, Switzerland), as described elsewhere [19]. Briefly, the system consists of an array of radio frequency antennas to be placed under the cage with group-housed, freely moving mice. The antennas would read the RFID tags and provide an approximate location of each animal with a time resolution of 50 ms. The series of RFID detection were exported and analyzed using custom routines developed in Matlab™ R2012b. The time interval during which an animal is detected constantly by the same antenna is defined as “visit”, and is described by time of first detection of a specific RFID and by its duration. When an animal is changing its location to a large enough extent to be detected by another antenna, a new visit is recorded. Visits that are longer than 10 min are considered inactive periods, consistent with resting/sleeping time (see also [19]). The number of visits was used as estimate of locomotor activity. The spontaneous locomotion is typically characterized by clusters of relatively short visits bounded by inactive periods, which we defined as “activity bouts”. A bout of activity is therefore described by the moment of appearance (for classification of occurrence in relation to the phases of the circadian cycle), the intensity of activity it comprises (number of visits); and the duration (defined as the time interval between the end of an inactive period, and the beginning of the next inactive period). Given that the spontaneous activity analyzed during the dark phase is part of a virtually infinite sequence, the number of bouts is equal to the number of inactive periods. In order to match the zebrafish data on spontaneous activity, we analyzed only the dark phase of the circadian cycle (when mice are normally active). In addition, we analyzed the novelty-induced hyperactivity in mice. Thus, we monitored the locomotor activity for 2 h after transferring the animals to a new cage with fresh bedding (otherwise identical to the home cage). The handling and the sudden change in the environment typically induces an abrupt increase in exploration/locomotor activity, which subsides within 2–3 h in group-housed animals (see also [19]). We assessed the habituation to a new environment by estimating the total locomotor activity and the IOC.

### Statistical analyses

All statistical analyses were performed using Statistica 10 software package (StatSoft Inc., Uppsala, Sweden). The data were analyzed by ANOVA (one-way, factorial, or repeated measures) as appropriate. Between-group differences were tested using post-hoc analysis: Dunnett's test for comparison against a single control group, as in one-way or repeated measures ANOVA design; and unequal N HSD test for comparisons against multiple controls as in simple factorial (“exposure” and “treatment” as factors) or between-group with replication factorial designs (“within-group” and “treatment” as factors). The ANOVA tables are shown in File S1. The threshold for statistical significance was set to 0.05 for all statistical analyses. All data are reported as average ± standard error of the mean.

## Results

### PFOS bioaccumulation in zebrafish larvae

The average concentration of PFOS measured in the zebrafish larvae was 21.6±5.4 ng/mg wet tissue and 213.5±62.7 ng/mg wet tissue in larvae exposed to 0.1 or 1 mg/L PFOS respectively. We did not find any effect of PFOS exposure on the viability, time of hatching, or incidence of developmental abnormalities (Table S1).

### Alterations in the pattern of spontaneous activity

The spontaneous swimming activity in zebrafish is characterized by discrete bouts of activity separated by inactive intervals. This pattern, also described as “beat-and glide”, is consistently displayed after the age of 4 dpf, and marks the developmental switch to increased spontaneous locomotion to support foraging [49]–[51]. However, although episodes of hyperactivity can be induced at 4 dpf (see [38]), the spontaneous activity is consistent over time and between individuals only at later stages, *i.e.* near the end of the yolk sac absorption and around the onset of the need for an external supply of energy [39], [52], [53]. The larvae exposed to 1 mg/L PFOS (Fig. 1 A) exhibit a dramatic disruption of the regular bout pattern, with a reduced frequency of activity bouts (Fig 1 B), but increased distance moved within a bout (Fig. 1 C). The shape and distribution of the activity bouts in larvae exposed to 0.1 mg/L PFOS was not different from controls (Fig. 1 B, C). Notably, the exposure to PFOS had a similar effect on the pattern of spontaneous locomotion in mice (Fig 1 D). We found a significant decrease in the number of spontaneous bouts of activity associated with an increase in the intensity of activity within the bout occurred only in the mice exposed to 3 mg/kg/day, but not in the 0.3 mg/kg/day PFOS-exposed mice (Fig. 1 E, F).

### Alterations in the VMR (zebrafish larvae) and novelty-induced hyperactivity (mice)

**Figure 2:**
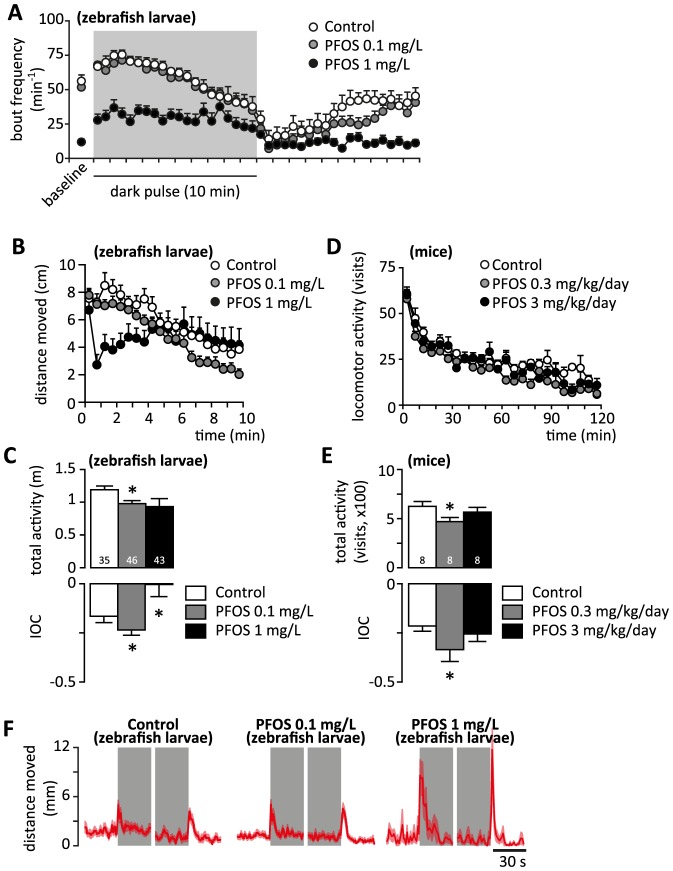
VMR in zebrafish larvae and novelty-induced hyperactivity in mice. (A) The dark pulse induces a similar pattern of fluctuations in the frequency of spontaneous bouts in larvae exposed to 0.1 mg/L PFOS as in controls. Larvae exposed to 1 mg/L PFOS respond with an increase in frequency that does not vary over time. In addition, the frequency of spontaneous bouts is restored directly to baseline level after the dark pulse. (B) Hyperactivity and habituation in zebrafish larvae during the dark pulse; 30 s timebins. (C) Quantification of total distance and IOC in zebrafish larvae. (D) Novelty-induced hyperactivity in mice. (E) Quantification of total distance moved and habituation rate (estimated by IOC) in mice. Note the similarity between the dose-response curves of the effect of exposure to PFOS on habituation in zebrafish and mice. (F) Swimming activity in zebrafish larvae at the transition between light and dark (gray shaded areas). The larvae exposed to 1 mg/L PFOS display hyperactive episodes of higher magnitude that last considerably longer than in controls. * p<0.05 PFOS exposed vs. control; ANOVA followed by Dunnett's post-hoc test. The number of independent observations is indicated at the bottom of each column in C and E. Graphs in A and B are based on the same number of observations reported in C; graphs in D are based on the same number of observations as in E.

The visual motor response (VMR) in zebrafish larvae describes the transient hyperactivity induced by a dark pulse during the light phase [36], [38], [52], [54]. Controls and larvae exposed to 0.1 mg/L PFOS, displayed an increase in the frequency of activity bouts (Fig 2 A) in addition to increasing the activity within the bout (Figure S2). In contrast, the larvae exposed to 1 mg/L PFOS appear to respond only with an increase in the number of bouts that is invariable over time (Fig 2 A). The transition from light to dark is typically associated with a robust increase in swimming activity that gradually decays over time as the larvae habituate to the environment (Fig. 2 B; see also [38], [54], [55] and Movie S2). The total distance moved during a 10 min dark pulse was lower, and the rate of habituation was higher (larger negative IOC value) as compared to controls only in the larvae exposed to 0.1 mg/L PFOS (Fig. 2 C). In light of these findings, we re-analyzed the data we reported earlier [19], focusing on the novelty-induced hyperactivity in mice (Fig. 2 D), and found that mice exposed to 0.3 mg/kg/day PFOS display both less locomotor activity, and faster habituation (larger negative IOC value) as compared to controls and mice exposed to 3 mg/kg/day PFOS (Fig. 2 E).

The VMR experimental setting also allows for investigating the effects of the transitions between light and dark phases [54]. The transition from light to dark induces a peak of activity (about 10 s) before stabilizing to a plateau-like level, consistently higher than the activity before the transition. In contrast, the transition from dark to light is characterized by a brief increase in swimming activity followed by an abrupt suppression of spontaneous swimming activity to a level not only lower than the activity before transition, but also lower than spontaneous activity before the dark pulse (Fig. 2 F). In zebrafish larvae exposed to 1 mg/L PFOS we found a stronger behavioral response to changes in light intensity. Following the transition from light to dark, the change in locomotor activity had a biphasic pattern, with an abrupt increase in activity, followed by a transient decrease before reaching the plateau level. At the transition from dark to light, the PFOS-exposed larvae displayed sharp peak of activity followed by a longer period of relatively high activity before settling at a level lower than at the end of the dark period (Fig 2 F). Importantly, the transition from dark to light has the electrophysiological features of a startle response (*e.g.* activation of Mauthner cells [36]). Therefore, we approached the startle response to a vibrational stimulus in order to refine the investigation of the altered swimming activity induced by PFOS.

### Alterations in the startle response

**Figure 3:**
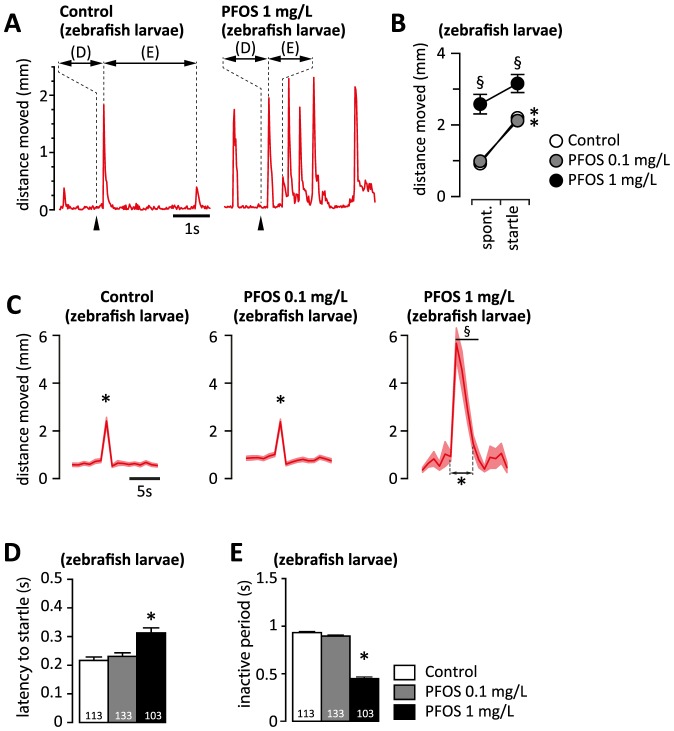
The effects of developmental exposure to PFOS on startle response in 6 dpf zebrafish larvae. (A) Typical vibrational startle response in a 6 dpf zebrafish larvae. Control larvae display a single bout of swimming activity after triggering the stimulus (arrowhead), followed by a prolonged silent period. Note that in control larvae, the swimming bout induced by the vibrational stimulus is considerably more robust than the spontaneous bouts (analysis shown in B), and is followed by a period of inactivity. In contrast, in larvae exposed to 1 mg/L PFOS, the vibrational stimulus is followed by a prolonged sequence of swimming bouts that have the amplitude similar to spontaneous bouts. The time interval between triggering the stimulus and the initiation of the bout is defined as latency to startle (analysis shown in D). The delay between the end of the startle bout and the following bout (analysis shown in E) can be defined as the latency to resume spontaneous swimming activity in control, but not in larvae exposed to 1 mg/L PFOS. (B) Comparison of the distance moved within spontaneous vs. stimulation-induced swimming bouts. Control larvae and larvae exposed to 0.1 mg/L PFOS consistently display a robust increase in the amplitude of activity bout in response to vibrational stimulation. In contrast, zebrafish larvae exposed to 1 mg/L PFOS swam significantly longer distances within spontaneous bout, but do not increase the distance moved in response to the vibrational stimulation. (C) Average distance moved integrated over 1s timebins. The increase in distance moved is accounted for by a single, more robust bout in controls (see A). In contrast, the amplitude of the startle response in larvae exposed to 1 mg/L PFOS is significantly larger than in controls, and it is presumably accounted for by more than 1 bout. In addition, the larvae exposed to 1 mg/L PFOS remain hyperactive for about 4 s after stimulation. The higher variability before in spontaneous activity before and after the startle response can be explained by irregularity in occurrence of spontaneous bouts. Note also that the larvae exposed to 1 mg/L PFOS display an increase in activity before the stimulation. (D, E) Analysis of the latency to startle (D) and inactive period (E). Zebrafish larvae exposed to 1 mg/L PFOS have longer latency to startle, and have shorter inactive period than controls or larvae exposed to 0.1 mg/L PFOS. B - repeated measures ANOVA followed by unequal N HSD post-hoc test; * p<0.05 startle vs. spontaneous; § p<0.05 PFOS exposed vs. controls. C –repeated measures ANOVA followed by unequal N HSD, or Dunnett's post-hoc test, respectively; § p<0.05 PFOS exposed vs. control; * p<0.05 vs. baseline. D, E - ANOVA followed by Dunnett's post-hoc test; * p<0.05 PFOS exposed vs. controls. The number of independent observations is indicated at the bottom of each column in D and E. The graphs in B and C are based on the same number of observation as reported in D and E.

The spontaneous activity of 6 dpf larvae in baseline conditions is characterized by bouts occurring at a rate of around 50/min, which yields a sub-unit probability to record 1 bout of activity within each second. Therefore, we inferred that measuring the distance moved by individual larvae in 1 s long timebins would accurately isolate the startle bout from the background spontaneous activity in control larvae. We found that the startle response in control larvae typically consists of a single bout of activity (Fig. 3 A) with the amplitude about 2.5 times larger than in spontaneous bouts (Fig. 3 B, C); the latency to startle is about 200 ms (Fig. 3 D), and the spontaneous swimming activity is resumed after an inactive period of about 1 s (Fig. 3 E). The startle response in larvae exposed to 0.1 mg/L PFOS was similar to controls. In contrast, the startle response in larvae exposed to 1 mg/L PFOS is characterized by a longer latency to startle, and is followed by a cluster of bouts of amplitude similar to the spontaneous bouts which are separated by very short inactive periods (Fig. 3 A–E). In addition, the larvae exposed to 1 mg/L PFOS also exhibit an increase in activity 1 s before stimulation (Fig. 3 C). Therefore, the startle response in control larvae can be described as an amplitude-modulation of spontaneous swimming bouts, while in PFOS-exposed larvae the startle response appears as frequency modulation of the locomotor output.

To summarize, the developmental exposure to 1 mg/L PFOS induced changes in spontaneous swimming activity at baseline characterized by less frequent, but more intense bouts of activity as compared to controls. In addition, stimuli that normally induce only a brief hyperactive episode in control larvae, in PFOS exposed larvae induce abnormally prolonged hyperactive episodes consisting of series of bouts of constant amplitude. This results in a pattern of spontaneous activity that is fragmented and highly variable over time (Figure S3). A similar pattern has been described recently in a zebrafish model aimed at investigating the developmental effects of knocking down Latrophilin 3 (Lphn3.1), an ADHD susceptibility gene, which was characterized by less numerous and misplaced dopaminergic neurons [56]. The phenotype was rescued by acute administration of drugs used in the treatment of ADHD [56]. Therefore, in light of the epidemiological report on the association between ADHD and the exposure to perflourinated chemicals (including PFOS) [16], we investigated whether the calming effect of dexamfetamine could be reproduced in our model.

### Effects of dexamfetamine and dopamine receptor agonists on spontaneous activity

**Figure 4:**
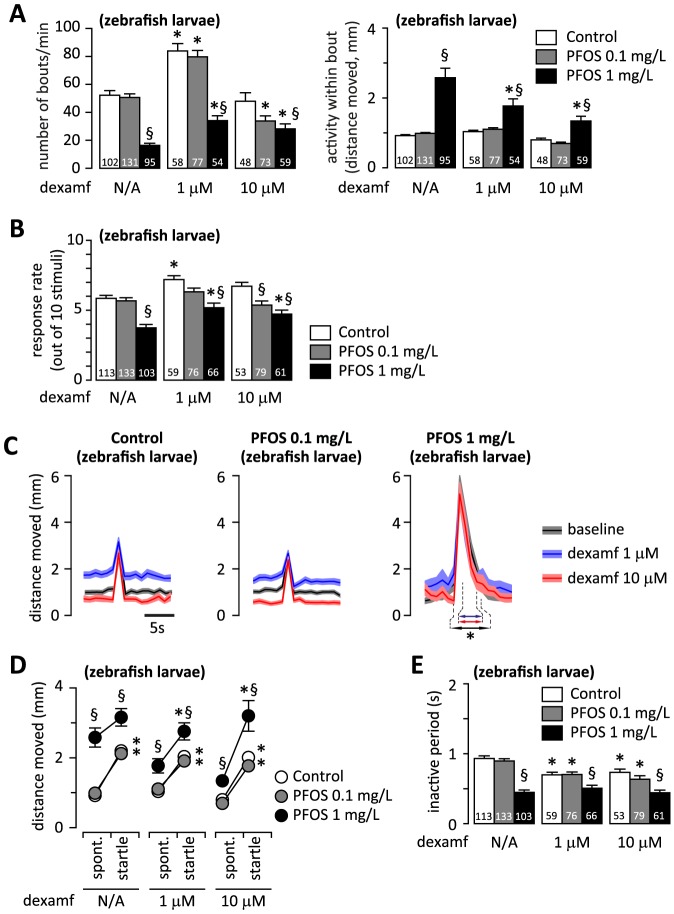
The effects of acute administration of dexamfetamine on spontaneous swimming and on startle response. (A) Dexamfetamine displays a bell-shaped dose-dependence of spontaneous bout frequency, but does not alter the distance swam per bout in controls and in larvae exposed to 0.1 mg/L PFOS. In contrast, dexamfetamine monotonically increases the frequency of spontaneous bouts of activity, and reduces the distance moved per bout in larvae exposed to 1 mg/L PFOS. (B) Average rate of response to vibrational stimulation. At baseline, the rate of response is significantly lower in the larvae exposed to 1 mg/L PFOS than in controls. Upon administration of dexamfetamine, the rate of response is increased only at 1 µM in controls, and at both doses in the larvae exposed to 1 mg/L PFOS. (C) Acute dexamfetamine administration alters the spontaneous activity before and after stimulation (presumably by altering the frequency of spontaneous bouts; see also A), but does not influence the amplitude of the startle response (accounted for by a single bout; see also D) in controls and larvae exposed to 0.1 mg/L PFOS. In larvae exposed to 1 mg/L PFOS, the amplitude of the startle response is not altered (presumably accounted for by more than one bout; see also D and E), but the duration of hyperactivity following the vibrational stimulation is shortened by both 1 and 10 µM dexamfetamine (see also Figure S5). (D) Dexamfetamine restores the amplitude-modulation of the activity bouts in larvae exposed to 1 mg/L PFOS by decreasing the distance moved for spontaneous bouts. (E) Dexamfetamine shortens the inactive period only in controls and in the larvae exposed to 0.1 mg/L PFOS. A, D - factorial ANOVA followed by unequal N HSD post-hoc test; § p<0.05 PFOS exposed vs. control; * p<0.05 vs. baseline. B- repeated measures ANOVA, followed by Dunnett's post hoc test with the first 3 samples as control (baseline); * p<0.05 vs. baseline. E - repeated measures ANOVA followed by unequal N HSD post-hoc test; * p<0.05 startle vs. spontaneous; § p<0.05 PFOS exposed vs. controls. The number of independent observations is indicated at the bottom of each column in A, B and E. Graphs in C and D are based on the same number of observation as reported in B and E.

We recorded the startle response in 6 dpf zebrafish larvae 30 min after spiking the rearing water with a catecholamine reuptake inhibitor, dexamfetamine (1 or 10 µM). The concentrations of dexamfetamine were selected based on the dose-response curves reported earlier [47], [48], according to which 1 µM should stimulate, while 10 µM should suppress spontaneous activity in control zebrafish larvae. We found that 1 µM dexamfetamine induced hyperactivity in controls and in larvae exposed to 0.1 mg/L PFOS by increasing the frequency of spontaneous activity bouts, but did not alter the distance moved within a bout (Fig 4 A). At 10 µM, dexamfetamine reduced the number of bouts of spontaneous activity only in the larvae exposed to 0.1 mg/L PFOS. In contrast, dexamfetamine monotonically increased the frequency of bouts of spontaneous activity and reduced the activity within the bout in the larvae exposed to 1 mg/L PFOS.

To confirm the dopamine signaling deficiency, and investigate the effects of dopamine receptor agonists on specific phenotypes, we treated the zebrafish larvae with non-specific (apomorphine), and specific (quinpirole for D2, and SKF-81297 for D1) dopamine receptor agonists. In zebrafish larvae exposed to 1 mg/L PFOS we found that quinpirole increased the frequency of spontaneous swimming bouts, while SKF-81297 reduced the within-bout activity (Figure S4). As expected, apomorphine treatment had effects consistent with the activation of both D1 and D2 receptors in larvae exposed to 1 mg/L PFOS (Figure S4).

### Effects of dexamfetamine and dopamine receptor agonists on the startle response

In controls, dexamfetamine increased the response rate (at 1 µM; Fig 4 B) and decreased the inactive period following the startle response (at both 1 and 10 µM; Fig 4 E), but did not alter the amplitude (Fig 4 C, D) or the latency (not shown) of the startle response. In larvae exposed to 0.1 mg/L PFOS, the only effect of dexamfetamine was a decrease in the inactive period (Fig 4 E. In larvae exposed to 1 mg/L PFOS, the increase in activity preceding the startle response was abolished by dexamfetamine (at both 1 and 10 µM; Fig. 4 C), and the duration of hyperactivity following the vibrational stimulus was reduced from 4.8 to 2.5 s (Fig 4. C, Figure S5). In addition, dexamfetamine monotonically increased the response rate (Fig 4 B), but had no effect on the latency (not shown) or the inactive period after the startle response (Fig 4 E) in larvae exposed to 1 mg/L PFOS.

The expected facilitating effect of dopamine receptor agonists on movement in the vibrational startle experiments manifested as a decrease in the latency to startle (induced by apomorphine), and in the inactive period (induced by apomorphine and quinpirole) in controls and larvae exposed to 0.1 mg/L PFOS (Figure S4). In larvae exposed to 1 mg/L PFOS, the dopamine receptor agonists had no effect on the parameters related to the startle response.

When we analyzed the amplitude of the activity bout induced by the vibrational stimulus in relation to the spontaneous activity bouts, we found that dexamfetamine treatment restored the amplitude modulation of the startle response in larvae exposed to 1 mg/L PFOS essentially by reducing the amplitude of spontaneous bouts (Fig 4 D).

## Discussion

In this study we found that zebrafish larvae exposed to PFOS in the rearing water from 2 hpf display dose-dependent behavioural alterations: zebrafish larvae exposed to 0.1 mg/L PFOS habituate faster than controls during a dark pulse, while 1 mg/L PFOS induces a disorganized pattern of spontaneous activity and persistent hyperactivity. Similarly, mice exposed to 0.3 mg/kg/day PFOS habituated faster than controls to a new environment, while mice exposed to 3 mg/kg/day PFOS displayed more intense and disorganized spontaneous activity. To further investigate the hyperactivity induced by PFOS, we analysed the startle response to a vibrational stimulus and found that larvae exposed to 1 mg/L PFOS display sustained hyperactivity after stimulation. Both spontaneous and startle-induced hyperactivity were partly reversed by acute administration of dexamfetamine.

In this study we found that zebrafish larvae exposed to PFOS in the rearing water from 2 to 144 hpf accumulated the chemical in the body, in agreement with earlier reported pharmacokinetics data [30]. Given that PFOS readily permeates the chorion [30], and that short-term exposure to PFOS did not induce detectable alterations (Figure S6), it is reasonable to assume that the behavioral effects are due to developmental damage, rather than to acute effects.

We found that the larvae exposed to 1 mg/L PFOS displayed disorganized spontaneous activity, characterized by less frequent, but more intense bouts. Similarly, mice prenatally exposed to 3 mg/kg/day PFOS displayed a decreased frequency associated with an increased intensity of spontaneous activity bouts. This pattern of alterations is suggestive of disturbances in the modulation of locomotion. To further investigate these alterations in zebrafish larvae, we induced sustained hyperactivity over a limited period by exposure to a dark pulse. The resulting fluctuations in distance moved was consistent with earlier reports and theoretical models [52], [55], [57]. The transition from light to dark induces an adaptive behavioral response that involves several regulatory circuits modulating spontaneous swimming [36]. In control larvae, the adaptation to dark consisted of a simultaneous modulation of bout frequency and activity within the bout. The monotonic decrease in swimming activity during the dark pulse is indicative of habituation [55], [58]. Interestingly, we found that the zebrafish larvae exposed to 0.1 mg/L PFOS did not display alterations in spontaneous activity, but habituated faster than controls. Similarly, mice exposed to PFOS at a dose that does not induce overt alterations in spontaneous locomotion were also found to habituate faster to a new environment. One can speculate that the defects in gating locomotion that result in a disorganized spontaneous activity pattern obscure the more subtle effect at motivational level. Alternatively, the different pattern of alterations in mice and zebrafish larvae may suggest that different mechanisms are responsible for the phenotype induced by developmental exposure to PFOS.

The larvae exposed to 1 mg/L PFOS displayed stereotypical adaptive changes in spontaneous activity with virtually no amplitude modulation of the activity bouts as compared to baseline, and very little variation in bout frequency during the dark pulse. The exaggerated response to the transition from light to dark was in agreement with the report by Huang et al [30]. In addition, we found that the larvae exposed to 1 mg/L PFOS displayed prolonged hyperactivity following the transition from dark to light. The behavioral modulation at the transition from dark to light shares kinematic and electrophysiological features with the startle response [36]. When we investigated the startle response to a vibrational stimulus, we found that the initial startle is followed by persistent hyperactivity in the larvae exposed to 1 mg/L PFOS. The rigidity in adaptation to changes in the environment is strongly suggestive of dopamine deficit, since it has been shown that dopamine signaling is required for behavioral variability [59], [60].

The developmental exposure to 1 mg/L PFOS induced complex phenotypical alterations consisting of a hypokinetic component associated to hyperactivity. The hypokinetic phenotype is illustrated by the reduced frequency of spontaneous activity bouts, while the hyperactivity is illustrated by the increase in distance moved within spontaneous activity bouts and by the persistent hyperactivity following the startle response. In light of the dopamine deficiency hypothesis, we tested whether dopamine receptors agonists can correct the behavioral alterations. Indeed, we found that the hypokinetic component was partly reversed by acute administration of D2 agonists (apomorphine and quinpirole), which also facilitated the initiation of movement in control larvae. The hyperactive phenotype was corrected by the D1 agonists (apomorphine and SKF-81297), which in turn had no effect in controls at the doses applied in our study. Importantly, the effects of dexamfetamine were more extensive than the effects of the dopamine receptor agonists, and corrected the hyperactive phenotype in a monotonic dose-dependent fashion. This suggests that the phenotype is caused by alterations in several diffuse neurotransmitter systems, such as noradrenaline and serotonin, in addition to the core defects in dopaminergic signaling. In zebrafish, the dopaminergic system is highly conserved [47], [61], [62], and plays an important role in regulating the swimming bouts in larvae [51]. A “hyperactive/impulsive motor phenotype” (consisting of clusters of very intense spontaneous activity) has been described recently in a zebrafish model established to investigate the effect of knocking down a homolog of Latrophilin 3 (Lphn3.1; one of the zebrafish orthologs of LPHN3, a susceptibility gene for ADHD [63]) during development [56]. The phenotype was reversed by acute administration of amfetamine-like psychostimulants commonly used for treating ADHD patients. In our study, the larvae exposed to 1 mg/L PFOS displayed clusters of intense spontaneous activity, as well as persistent hyperactivity following the startle response. In addition, the calming effect of dexamfetamine was replicated in larvae exposed to 1 mg/L PFOS, as illustrated by the dramatic correction of both spontaneous, and induced hyperactivity.

Several lines of evidence support the association of ADHD with alterations in dopamine signaling (reviewed in [64], [65]). The core alteration behind the hyperactivity and impulsivity has been suggested to be a deficit in behavioral inhibition [66], [67]. Impulsivity due to reduced behavioral inhibition has been shown to correlate with serum concentrations of PFOS in children independently from an actual diagnosis of ADHD [15]. In addition, the relative risk of being diagnosed with ADHD was found to increase with the level of PFOS in the peripheral blood [16]. An important limitation of the cross-sectional studies [15], [16] is the unavailability of data on maternal exposure during pregnancy. Nevertheless, the age range in the study cohorts place the birth of the children in the early 1990's, a period corresponding to the peak in PFOS exposure in the general population [12], [68]. Therefore, one can speculate that an early (developmental) exposure is very likely to have contributed to the outcomes analyzed in the cross-sectional studies. In the case of PFCs, an important source of exposure used to be the grease-resistant packaging [7] used for food with high fat content, such as fast-food products, chips and snacks, which in itself is documented to increase the risk of ADHD [69]. Here we show that in zebrafish exposure to PFOS during development induces hyperactivity and a dopaminergic signaling deficit that can be corrected by acute administration of dexamfetamine. Mice exposed to PFOS *in utero* exhibit similar alterations and dose-response curve. In conclusion, both experimental models show that exposure to PFOS during development induces core features of the phenotype observed in PFOS-exposed children.

## Supporting Information

Figure S1 **Analysis of relationships among the variables selected for analysis.** (A) Loading plot for the first two factors extracted by means of PLS-DA (projection to latent structure – discriminant analysis) (see also [70]). The model included variables from both spontaneous activity (denoted by “r” in the beginning of the variable name) and startle response (denoted by “s” in the beginning of the variable name) in larvae that were not subject to pharmacological manipulations. Variables projecting symmetrically relative to the origin of the axes may be negatively correlated, while variables mapped very close together may be positively correlated. Note that the spontaneous activity within the bout (r_dist/bout) and the spontaneous bout frequency (r_bouts/min) mapped symmetrically relative to the origin of the axes, but the negative correlation was not confirmed. (B) It is obvious that the developmental exposure to PFOS 1 mg/L had significant influence on both variables, which rendered the multivariate approach uninformative. The most important information provided by the multivariate model above was that the variables we selected were orthogonal to a large-enough extent to justify their consideration as independent variables analysed separately in multifactorial models.
(EPS)

Figure S2 **Analysis of hyperactivity induced by a dark pulse in 6 dpf zebrafish larvae (30 s timebins).** The total distance moved within the bout does not differ between baseline and the dark period, but decreases during the dark period. The sudden increase at the transition between dark and light is due to the startle-like response (see text for details). The distance moved per bout drops after the transition from dark to light in controls and larvae exposed to 0.1 mg/L PFOS. In contrast, these fluctuations cannot be identified in larvae exposed to 1 mg/l PFOS, possibly because of the disorganized pattern of spontaneous activity (very high within-individual variations). Note, though, the dramatic variations at the transitions between light and dark phases in larvae exposed to 1 mg/L PFOS. The pattern of changes in the activity during the VMR testing is consistent with earlier reports and theoretical models [52], [55], [57]. In our experiment, the larvae had not been exposed to any dark period before recording the swimming activity. Therefore the spontaneous activity at baseline was constant and did not show significant long-term trends (not shown). In line with the analysis of the startle response, the amplitude modulation of the activity bouts is absent in the larvae exposed to 1 mg/l PFOS. Yet, the total distance swam during the 10 min dark pulse is not different from controls. Therefore, in agreement with earlier reports [39], several orthogonal parameters should be used for characterizing the swimming phenotype in zebrafish larvae. In addition, we argue that the characterization of alterations induced by exposure to potentially neurotoxic compounds in animal models must include the analysis of spontaneous activity alongside with induced behavioural responses.
(EPS)

Figure S3 **Illustrative individual traces of spontaneous activity over 1 min (3 s timebins).** Note the heavily fragmented pattern in the larvae exposed to 1 mg/l PFOS (right) as compared to controls (left). A similar pattern, described as “hyperactive/impulsive motor phenotype”, was found in a zebrafish model investigating the role of Latrophilin 3 (Lphn3.1) in the etiology of ADHD [56].
(EPS)

Figure S4 **Synoptic illustration of the effects of dopamine receptors agonists and D-amfetamine on activity bouts in 6 dpf zebrafish larvae.** (A, B) Effects on spontaneous activity. Note that quinpirole (D2 receptor agonist) increases the frequency of bouts, but does not alter the activity within the bout, while SKF-81297 (D1 receptor agonist) reduces the activity within the bout, but does not change the frequency of spontaneous bouts. As expected, apomorphine (nonspecific dopamine receptor agonist) has an effect that shares features of both D1 and D2 receptor agonists. (C, D) Effects on the startle response. Note that the latency to startle in controls is affected only by apomorphine. Otherwise, dopamine receptors agonists have no effect at the doses we tested. In contrast, the inactive period is shortened by all compounds, except the D1 agonist SKF-81297. Neither the latency to startle, nor the inactive period are affected by any of the compounds in the larvae exposed to 1 mg/L PFOS. Factorial ANOVA followed by unequal N HSD post-hoc test; § p<0.05 PFOS exposed vs. control; * p<0.05 vs. baseline.
(EPS)

Figure S5 **The effect of dexamfetamine on the startle-induced hyperactivity (SIH) in zebrafish larvae exposed to 1 mg/L PFOS.** (A) SIH consists of a cluster of bouts of activity separated by short inactive periods (see also the main text for details). To estimate the duration of SIH, we measured the delay between the beginning of the startle response and the first occurrence of an inactive period longer than 0.7 s (i.e. 1 standard deviation longer than the average first inactive period following the startle response; see Fig 3E). The arrowhead indicates the moment when the stimulus was triggered. (B) Acute administration of dexamfetamine consistenly shortens the delay of occurrence of inactive periods longer than 0.7 s. One-way ANOVA followed by Dunnett's post-hoc test; * p<0.05 vs. baseline. The analysis described above is not applicable to controls or to larvae exposed to 0.1 mg/L PFOS because the inactive period following the startle response is not significantly different from the average delay between spontaneous bouts (see Fig 1B, 3E, 4A and 4E). In contrast, spontaneous bouts in zebrafish larvae exposed to 1 mg/L PFOS occur on average every 6 s at baseline, and every 1.5–2 s after dexamfetamine treatment (see Fig 4A). Neither apomorphine, nor specific dopamine receptor agonists had significant effects on SIH duration (not shown).
(EPS)

Figure S6 **The effects of short-term exposure to PFOS 1 mg/L.** Zebrafish larvae were exposed to 0.1 or 1 mg/L PFOS for 24 h before recording the VMR at 6 dpf. Control larvae received an equivalent amount of vehicle (DMSO). The final concentration of DMSO in the E3 water was 0.1%. (A) Distance moved in 30 s timebins before (baseline), during, and after a 10 min dark pulse. (B) Swimming activity at the transition between light and dark (gray shaded areas). The short-term exposure to PFOS had no significant effect on either distance moved, or reaction to sudden changes in light intensity.
(EPS)

Movie S1 **Slow motion (0.05x) of a normal startle response recorded in 6 dpf zebrafish larvae.** The light marks the moment of triggering the acoustic stimulus. Note that the fish initiates a brief sequence of swimming motions with a certain delay after the application of the stimulus and moves about 2 mm away from the initial position. Recorded at 100 fps; well diameter: 10 mm.
(AVI)

Movie S2 **Visual motor response (VMR) in 6 dpf zebrafish larvae.** The transition from light to dark (see marker at the bottom) induces a hyperactive reaction in the larvae. Note that the control larvae (top row) react almost instantaneously after turning the transition to dark, while the larvae exposed to 1 mg/L PFOS (bottom row) have a delayed, but exaggerated response. Recorded at 50 fps; well diameter: 10 mm.
(AVI)

Table S1 **Embryonal mortality and malformation rates in zebrafish larvae exposed to 0.1 or 1 mg/L PFOS from 2 hpf until the timepoint for analysis.**
(DOCX)

File S1 **ANOVA tables for the data shown in the main figures.**
(DOCX)
