# Supplementary material for: PFOS Induces Behavioral Alterations, Including Spontaneous Hyperactivity That Is Corrected by Dexamfetamine in Zebrafish Larvae
Source: PLoS One. 2014 Apr 16;9(4):e94227. doi: 10.1371/journal.pone.0094227 (PMC3989208; doi:10.1371/journal.pone.0094227)
Supplement: Table S1 — Embryonal mortality and malformation rates in zebrafish larvae exposed to 0.1 or 1 mg/L PFOS from 2 hpf until the timepoint for analysis. (DOCX) [file pone.0094227.s009.docx]

**Supplementary Table 1**

Embryonal mortality and malformation rates in zebrafish larvae exposed to 0.1 or 1 mg/L PFOS from 2 hpf

| treatment | mortality | | | embryonal defects | total |
| --- | --- | --- | --- | --- | --- |
|  | 24 hpf | 48 hpf | 144 hpf | 144 hpf |  |
| CTRL (0.01% DMSO) | 3.6% | 4.1% | 4.5% | 1.4% | 222 |
| PFOS 0.1 mg/L | 3.7% | 4.6% | 5.0% | 2.3% | 219 |
| PFOS 1 mg/L | 2.3% | 4.6% | 5.0% | 3.2% | 219 |
