# Supplementary material for: PFOS Induces Behavioral Alterations, Including Spontaneous Hyperactivity That Is Corrected by Dexamfetamine in Zebrafish Larvae
Source: PLoS One. 2014 Apr 16;9(4):e94227. doi: 10.1371/journal.pone.0094227 (PMC3989208; doi:10.1371/journal.pone.0094227)
Supplement: File S1 — ANOVA tables for the data shown in the main figures. (DOCX) [file pone.0094227.s010.docx]

**Multifactorial analyses**

**Fig. 2F** Transient hyperactivity induced in zebrafish larvae by the sudden change in light intensity. Between-group with replication (repeated measures ANOVA) design.

Transition from light to dark

| Effect | SS | df | MS | F | p |
| --- | --- | --- | --- | --- | --- |
| Intercept | 12046.10 | 1 | 12046.10 | 242.754 | 0.000 |
| PFOS | 226.23 | 2 | 113.12 | 2.280 | 0.110 |
| Error | 3374.34 | 68 | 49.62 |  |  |
| replication | 3960.26 | 60 | 66.00 | 11.888 | 0.000 |
| PFOS*replication | 1632.24 | 120 | 13.60 | 2.450 | 0.000 |
| Error | 22652.30 | 4080 | 5.55 |  |  |

Transition from dark to light

| Effect | SS | df | MS | F | p |
| --- | --- | --- | --- | --- | --- |
| Intercept | 4904.23 | 1 | 4904.229 | 189.093 | 0.000 |
| PFOS | 31.70 | 2 | 15.850 | 0.611 | 0.546 |
| Error | 1763.62 | 68 | 25.936 |  |  |
| replication | 4115.76 | 59 | 69.759 | 17.213 | 0.000 |
| PFOS*replication | 1117.10 | 118 | 9.467 | 2.336 | 0.000 |
| Error | 16259.61 | 4012 | 4.053 |  |  |

**Fig. 3B** Comparison between spontaneous and startle-induced activity bouts. Between-group with replication (repeated measures ANOVA) design.

| Effect | SS | df | MS | F | p |
| --- | --- | --- | --- | --- | --- |
| Intercept | 2216.756 | 1 | 2216.756 | 700.134 | 0.000 |
| PFOS | 167.074 | 2 | 83.537 | 26.384 | 0.000 |
| Error | 972.020 | 307 | 3.166 |  |  |
| replication | 116.563 | 1 | 116.563 | 57.648 | 0.000 |
| PFOS*replication | 19.306 | 2 | 9.653 | 4.774 | 0.009 |
| Error | 620.744 | 307 | 2.022 |  |  |

**Fig. 3C** The effect of developmental exposure to PFOS on startle-induced hyperactivity in zebrafish larvae. Between-group with replication (repeated measures ANOVA) design.

| Effect | SS | df | MS | F | p |
| --- | --- | --- | --- | --- | --- |
| Intercept | 8831.80 | 1 | 8831.800 | 502.782 | 0.000 |
| PFOS | 206.70 | 2 | 103.352 | 5.884 | 0.003 |
| Error | 6077.79 | 346 | 17.566 |  |  |
| replication | 2446.19 | 15 | 163.079 | 77.642 | 0.000 |
| PFOS*replication | 1341.00 | 30 | 44.700 | 21.282 | 0.000 |
| Error | 10901.04 | 5190 | 2.100 |  |  |

Dunnett’s post-hoc test for replication effect; reference set to baseline activity (5s before triggering the stimulus); stimulus triggered immediately after timepoint “0s”.

|  | DMSO | PFOS 0.1 | PFOS 1 |
| --- | --- | --- | --- |
| -5s | N/A | N/A | N/A |
| -4s | 0.851 | 0.949 | 0.921 |
| -3s | 0.705 | 0.708 | 0.766 |
| -2s | 0.811 | 0.709 | 0.715 |
| -1s | 0.250 | 0.816 | 0.242 |
| 0s | 0.064 | 0.045 | 0.038 |
| 1s | 0.000 | 0.000 | 0.000 |
| 2s | 0.955 | 0.998 | 0.000 |
| 3s | 0.508 | 0.986 | 0.000 |
| 4s | 0.701 | 0.982 | 0.003 |
| 5s | 0.502 | 0.966 | 0.187 |
| 6s | 0.357 | 0.994 | 0.638 |
| 7s | 0.746 | 0.994 | 0.638 |
| 8s | 0.408 | 0.984 | 0.714 |
| 9s | 0.837 | 0.990 | 0.471 |
| 10s | 0.715 | 1.000 | 0.766 |

**Fig. 4A** The effect of dexamfetamine on spontaneous activity in zebrafish larvae exposed to PFOS during development. Simple factorial (ANOVA) design.

Spontaneous bout frequency

| Effect | SS | df | MS | F | p |
| --- | --- | --- | --- | --- | --- |
| Intercept | 1382068 | 1 | 1382068 | 1268.925 | 0.000 |
| PFOS | 141741 | 2 | 70871 | 65.069 | 0.000 |
| dexamfetamine | 102870 | 2 | 51435 | 47.224 | 0.000 |
| dexamfetamine*PFOS | 32036 | 4 | 8009 | 7.353 | 0.000 |
| Error | 724294 | 665 | 1089 |  |  |

Activity within the bout

| Effect | SS | df | MS | F | p |
| --- | --- | --- | --- | --- | --- |
| Intercept | 973.448 | 1 | 973.448 | 679.334 | 0.000 |
| PFOS | 35.013 | 2 | 17.507 | 12.217 | 0.000 |
| dexamfetamine | 125.098 | 2 | 62.549 | 43.651 | 0.000 |
| dexamfetamine*PFOS | 32.882 | 4 | 8.221 | 5.737 | 0.000 |
| Error | 952.908 | 665 | 1.433 |  |  |

**Fig. 4B** The effect of dexamfetamine on the probability to mount a startle response in zebrafish larvae exposed to PFOS during development. Simple factorial (ANOVA) design.

| Effect | SS | df | MS | F | p |
| --- | --- | --- | --- | --- | --- |
| Intercept | 214.025 | 1 | 214.025 | 3433.849 | 0.000 |
| PFOS | 4.264 | 2 | 2.132 | 34.210 | 0.000 |
| dexamfetamine | 1.900 | 2 | 0.950 | 15.243 | 0.000 |
| dexamfetamine* PFOS | 0.529 | 4 | 0.132 | 2.124 | 0.076 |
| Error | 45.749 | 734 | 0.062 |  |  |

**Fig. 4C** The effect of dexamfetamine treatment on startle response - an illustration of the effect addressed in Supplementary Fig. 5.

**Fig. 4D** The effect of dexamfetamine on the difference between spontaneous bouts, and bouts triggered by vibratory stimulation in zebrafish larvae exposed to PFOS during development. Between-group with replication (repeated measures ANOVA) design.

| Effect | SS | df | MS | F | p |
| --- | --- | --- | --- | --- | --- |
| Intercept | 3918.822 | 1 | 3918.822 | 1593.182 | 0.000 |
| PFOS | 241.891 | 2 | 120.945 | 49.170 | 0.000 |
| dexamfetamine | 15.741 | 2 | 7.870 | 3.200 | 0.041 |
| dexamfetamine* PFOS | 9.268 | 4 | 2.317 | 0.942 | 0.439 |
| Error | 1630.811 | 663 | 2.460 |  |  |
| replication | 367.024 | 1 | 367.024 | 191.947 | 0.000 |
| PFOS*replication | 1.695 | 2 | 0.848 | 0.443 | 0.642 |
| dexamfetamine*replication | 18.887 | 2 | 9.444 | 4.939 | 0.007 |
| dexamfetamine*PFOS*replication | 30.567 | 4 | 7.642 | 3.996 | 0.003 |
| Error | 1267.728 | 663 | 1.912 |  |  |

We then investigated the effects of each dose of dexamfetamine independently.

Dexamfetamine 1 µM

| Effect | | SS | | df | MS | | | F | | p |  |
| --- | --- | --- | --- | --- | --- | --- | --- | --- | --- | --- | --- |
| Intercept | | 1163.717 | | 1 | 1163.717 | | | 875.528 | | 0.000 |  |
| PFOS | | 46.456 | | 2 | 23.228 | | | 17.476 | | 0.000 |  |
| Error | | 243.236 | | 183 | 1.329 | | |  | |  |  |
| replication | | 82.314 | | 1 | 82.314 | | | 65.207 | | 0.000 |  |
| PFOS*replication | | 1.291 | | 2 | 0.645 | | | 0.511 | | 0.601 |  |
| Error | 231.011 | | 183 | | | 1.262 |  | |  | | |

Dexamfetamine 10 µM

| Effect | SS | df | MS | F | p |
| --- | --- | --- | --- | --- | --- |
| Intercept | 934.9192 | 1 | 934.9192 | 389.217 | 0.000 |
| PFOS | 68.7118 | 2 | 34.3559 | 14.303 | 0.000 |
| Error | 415.5548 | 173 | 2.4021 |  |  |
| replication | 179.7357 | 1 | 179.7357 | 74.751 | 0.000 |
| PFOS*replication | 12.2233 | 2 | 6.1116 | 2.542 | 0.082 |
| Error | 415.9731 | 173 | 2.4045 |  |  |

Note that the interaction (PFOS*replication) effect disappears at 1 µM dexamfetamine, indicating that the amplitude-modulation of the startle response is restored in the larvae exposed to 1 mg/L PFOS. The interaction effect approaches significance at 1 µM dexamfetamine presumably because of the dramatic change in spontaneous activity (which greatly amplifies the difference between spontaneous and startle-induced bouts in the larvae exposed to 1 mg/L PFOS).

**Fig. 4E** The effect of dexamfetamine on the duration of the inactive period following the startle response in zebrafish larvae exposed to PFOS during development. Simple factorial (ANOVA) design. Note that in control larvae, the inactive period represents the latency to resume spontaneous activity, while in larvae exposed to 1 mg/L PFOS it represents the delay between the initial bout triggered by the vibratory stimulus and the following bout in the cluster (see also the comment on Supplementary Figure 4).

| Effect | SS | df | MS | F | p |
| --- | --- | --- | --- | --- | --- |
| Intercept | 288.300 | 1 | 288.300 | 2143.022 | 0.000 |
| PFOS | 14.086 | 2 | 7.043 | 52.353 | 0.000 |
| dexamfetamine | 2.873 | 2 | 1.436 | 10.677 | 0.000 |
| dexamfetamine* PFOS | 3.304 | 4 | 0.826 | 6.140 | 0.000 |
| Error | 94.709 | 704 | 0.135 |  |  |
